# Implementing a manual dashboard for blood center management in under-resourced settings

**DOI:** 10.6026/973206300221732

**Published:** 2026-03-31

**Authors:** Sanjay Kumar Thakur, Arun Kumar, Mohd Fazil, Santosh Kumar Sharma, Lokesh Kumar, Arzoo Jahan, Ruchika Gupta, Sompal Singh

**Affiliations:** 1 Department of Blood Bank, Regional Blood Transfusion Centre, Hindu Rao Hospital and NDMC Medical College, North Delhi 110007, Delhi, India; 2 Department of Pathology, Hindu Rao Hospital and NDMC Medical College, North Delhi 110007, Delhi, India; 3 Department of Cytopathology, ICMR-National Institute of Cancer Prevention and Research, Noida 201301, Uttar Pradesh, India

**Keywords:** Dashboard, key performance indicators (KPIs), quality management systems (QMS)

## Abstract

Blood transfusion is vital in healthcare, aiding surgeries, traumatic injuries, and chronic conditions. Enhancements in Transfusion Medicine focus on safe blood products and effective Quality Management Systems (QMS) to minimize transfusion risks. Evaluating key performance indicators (KPIs) is crucial for quality assurance and ongoing improvement in blood transfusion services. Therefore, it is of interest to evaluate a healthcare facility's compliance with NABH standards for blood transfusion services using 11 Key Performance Indicators (KPIs). Findings show Transfusion Transmissible Infections (TTIs) consistently below benchmarks at an average of 2.19%, with all parameters except Syphilis meeting standards. The Adverse Transfusion Reaction Rate (ATRR) averaged 0.36%, indicating robust safety protocols, while blood wastage rates showed significant variation, highlighting the need for better inventory management. Turnaround times for cross-matches met benchmarks, averaging 27.29 minutes for emergencies and delayed transfusions stayed under 15%. Overall, the study highlights areas for improvement, particularly in wastage rates and donor management, underscoring the necessity for ongoing quality assessment and continuous improvement initiatives to enhance patient safety and care quality in transfusion practices. KPI-Dashboard is very helpful in identifying the current lacunae which can be very helpful in improving blood transfusion services. Present study describes a concept of manual dashboard.

## Background:

Blood transfusion plays a crucial role in modern healthcare, providing life-saving support to patients undergoing surgeries, managing traumatic injuries or coping with chronic medical conditions. The pursuit of safe blood products has led to significant growth in transfusion medicine, emphasizing the importance of robust Quality Management Systems (QMS) to achieve near zero-risk transfusions. The evaluation of KPIs within blood transfusion services is essential for quality assurance and continuous improvement. Scrutinizing parameters such as Transfusion Transmissible Infections (TTIs), blood component preparation, adverse transfusion reactions and turnaround time for cross- matching provides valuable insights into clinical practices and adherence to standards [1]. Ensuring the right blood for the right patient at the right time and place is fundamental, necessitating the selection of appropriate, high-quality blood components [2]. Healthcare institutions worldwide adhere to stringent quality standards set by accreditation bodies such as AABB (American Association of Blood Bank), CAP (College of American Pathologists), ISBT (International Society of Blood Transfusion) and JPAC (Joint United Kingdom Blood Transfusion Services Professional Advisory Committee) [3]. These bodies monitor transfusion service quality using Quality Indicators (QI) or Key Performance Indicators (KPI). Continuous monitoring and the application of Corrective and Preventive Actions (CAPA) are vital for ensuring the efficacy of QMS [4]. In India, National Accreditation Board for Hospitals & Healthcare Providers (NABH) and National Accreditation Board for Testing and Calibration Laboratories (NABL) oversee accreditation, enforcing standards aligned with statutory regulations and guidelines [5]. While not mandatory, many blood centers in India seek NABH accreditation, which sets standards and suggests KPIs to facilitate continual improvement, ultimately ensuring blood safety from donor vein to patient vein [6, 7].

Benchmarking is a cornerstone process in organizational enhancement, involving systematic comparisons against industry exemplars. This perpetual assessment relies on benchmarks to identify areas for refinement, contingent upon an organization's openness to change and readiness to adopt emerging best practices. The objectives of benchmarking are diverse, aiming to enhance operational processes, refine products or services and augment overall customer satisfaction. This endeavor unfolds through a structured process, with the identification of KPIs being integral. Realistic, specific, measurable and quantifiable KPIs are essential for gauging progress and aligning operations with strategic goals. Regular collection and review of KPIs tailored to individual laboratory needs foster continual improvement and prompt corrective action when deviations occur. Dashboards offer real-time visualization of KPIs against benchmarks, aiding in trend identification and operational optimization [8]. In clinical laboratory settings, KPIs traverse pre-analytical, analytical and post-analytical phases, addressing crucial performance facets [8, 9]. Furthermore, Management Review Meetings (MRMs) provide forums for evaluating organizational performance and identifying improvement opportunities. These meetings delve into various aspects including audit results, customer feedback and risk management, fostering accountability and driving continual improvement [10]. Woo *et al.* elucidated the efficacy of integrating a real-time Web-based dashboard into the transfusion service for managing blood product inventory and enhancing workflow [11]. Therefore, it is of interest to report actionable insights for healthcare providers and policymakers to optimize patient outcomes and ensure the delivery of high-quality, safe and efficient blood transfusion services.

## Materials and Methods:

## Ethical committee clearance:

The Institutional Ethical Committee granted approval by letter No F.No: IEC/NDMC/2021/69.

## Study design:

Present study is an observational study and performed from January 2023 to December 2023 at the Regional Blood Transfusion Centre (RBTC).

## Study participants:

All the data of blood donation, blood component preparation, TTIs testing, quality control, blood component demand and blood component issued to the recipient maintained as per records in the inventory registers of different sections of the department during the study period, was obtained from the various sections of the department for this study. Benchmarks used in this study were taken from previous studies.

## Study procedure:

NABH has outlined ten quality indicators, which are recommended for data collection by all blood banks [1]. Additionally, NABH accredited blood banks are mandated to monitor and report the data for the first five of these indicators to NABH every six months.

## Calculation of KPIs:

## Percentage of Transfusion transmissible infection (TTI %):

The study data for total number of donation and TTIs reactive blood units was obtained from the master register and TTIs testing register on monthly basis for HIV, HBV, HCV, syphilis and malaria parasite (MP). The KPI for Percentage of total transfusion transmissible infection positive (TTI %) and similar for each TTIs (HIV, HBV, HCV, syphilis malaria) was obtained by using the following formula:

TTI%=(units reactive for one or more TTI (HIV + HBV + HCV + Syphilis + Malaria))/(Total no.of Donations) X 100

HIV %=(Reactive reactive units for HIV)/(Total no.of Donations) X 100

HBV %=(Reactive reactive units for HBV)/(Total no.of Donations) X 100

HCV %=(Reactive reactive units for HCV)/(Total no.of Donations) X 100

Syphilis %=(Reactive reactive units for Syphilis)/(Total no.of Donations) X 100

MP%=(Reactive reactive units for MP)/(Total no.of Donations) X 100

## Adverse transfusion reaction rate (ATRR %):

The study data for number of adverse transfusion reaction was obtained from transfusion reaction register and the record of total number of blood component issued was obtained from blood components issue registers on monthly basis. The percentage of adverse transfusion reaction rate was calculated using the following formula:

ATRR%:=(Total no.of adverse transfusion reactions)/(Total no.of blood components issued) X 100

## The wastage rate% (excluding discard due to TTIs reactivity):

The study data for blood component wastage due to various reasons like; breakage/leakage, under collection, hemolyzed, lipemic, high bilirubin, units used for quality control, excluding discard due to TTI reactivity. The data was obtained from blood component discard registers. Record of total number of blood components preparation was obtained from blood component preparation register. The wastage rate% (excluding discard due to TTIs reactivity) was calculated using the following formulas:

Wastage rate%=(No.of blood/blood components discarded)/(Total no of blood/blood components prepared) X100

WB+PRBC Wastage rate%=(No.of WB+PRBC discarded)/(No.of WB collected+PRBC prepared) X 100

RDP Wastage rate%=(No.of RDP discarded)/(No.of RDP prepared) X 100

FFP Wastage rate%=(No.of FFP discarded)/(No.of FFP prepared) X 100

## Turnaround time for cross-match/blood component issue (TAT):

The study data for turnaround time of blood component issue (TAT) was obtained from blood /blood component request form, blood request receiving register and cross-match register. The duration measured from the moment the blood request with sample received at reception counter in the blood center until the blood is ready to issue after cross-matching. The TAT of urgent / emergency requests for blood component and routine / elective cases of cross-match were calculated separately. The cases of blood group discrepancy or cross match incompatibility that required additional special technique were excluded from this study. A non-probabilistic sample of all emergency blood component issued during the study period was used to calculate Turnaround Time (TAT). The fifth emergency and routine request in the morning shift, the fifth emergency and routine request in the afternoon shift and the fourth and seventh request in the night shift were included in the study for calculation of TAT. The TAT was calculated using the following formulas:

TAT of cross-match=(Sum of the time taken for crossmatch)/(Total number of blood and blood components crossmatched/reserved)

## Component QC failures (for each component):

The data of blood component QC failure were obtained from Quality Control registers of each component; whole blood (WB), packed red blood cells (PRBC), random donor platelets (RDP) and fresh frozen plasma (FFP). At least 1% units of the each component were tested for Quality Control, out of which 75% should match the acceptable ranges. The percentage of component QC failures was calculated using the following formulas:

WB QC failures%=(No.of WB QC failures)/(Total no.of WB tested) X 100

PRBC QC failures%=(No.of PRBC QC failures)/(Total no.of PRBC tested) X 100

RDP QC failures%=(No.of RDP QC failures)/(Total no.of RDP tested) X 100

FFP QC failures%=(No.of FFP QC failures)/(Total no.of FFP tested) X 100

## Adverse donor reaction rate (%):

The data for calculation of adverse donor reaction rate was obtained from adverse donor reaction register and bleeding (blood donation) register and calculation was performed using formula below:

Adverse Donor Reaction Rate%=(No.of donors experienced adverse reaction)/(Total number of donors ) X 100

## Donor deferral rate (%):

The data for calculation of donor deferral rate (%) was obtained from donor deferral register and blood donation register and calculation was performed using formula below:

Donor Deferral Rate%=(Number of donor deferrals)/(Total no.of donation + total no.of deferrals) X 100

## The percentage of component issues:

The data for calculation of percentage of components issued was obtained from blood component issue registers and calculation was performed using formula below:

Component issue %=(Total component issues)/(Total WB issue+Total component (PRBC+RDP+FFP) issues) X 100

## TTI outlier's %:

Control charts (L.J. Chart) for HIV, HBV and HCV were prepared and no. of deviations beyond ±2SD (outliers) were identified. Calculation for HIV, HBV and HCV was performed using formula below:

TTI outlier's %=(Number of deviations beyond ±2SD)/(Total Nuber of batches asseys ) X 100

## Delay in transfusion beyond 30 min after issue:

This delay in transfusion beyond 30 min after issue calculated by employing a non-probabilistic sampling method by choosing the 3rd and 5th issues in the morning shift, 5th issue in the afternoon shift and 5th issue in the night shift during the study period. The absolute number of such delays noted.

## Total blood component issue to blood collection (donation ratio):

The data for total blood component issue was obtained from component issue register and bleeding (blood donation) register and calculation was performed using formula below:

Total blood component issue to blood collection = (Total no.of blood component issue)/(Total number of collection)

## Results:

In this study, we evaluated various key performance indicators (KPIs) to assess the compliance of a healthcare facility with the NABH standards. The observations were recorded monthly throughout the year 2023 and the results are illustrated in Table 1 (see PDF). Summary of poorly performing key performance indicators (KPI), problem identified and action taken to achieve benchmark are illustrated in Table 2 (see PDF). Throughout 2023, the prevalence of TTIs, including HIV, HBsAg, HCV, syphilis and malaria, ranged from 1.10% to 2.65%, with an annual average of 2.19%. The annual percentage of HIV, HBsAg, HCV, syphilis and malaria was 0.25%, 0.97%, 0.43%, o.55% and 0.00% respectively. All TTIs remained consistently below the benchmark except syphilis, indicating successful pre-donation donor assessment and counseling. The ATRR remained below the benchmark. The ATRR remained consistently below the benchmark of 2%, with an annual average of 0.36% and no hemolytic transfusion reaction (0.00%) was observed during the study period, reflecting effective safety measures in blood transfusion practices. The wastage rate of blood and blood products, excluding TTIs-reactive units, varied from 1.86% (October) to 23.58% (January), with an overall 11.48% annually. Wastage rates of WB, varied from 0.00% (August) to 3.56% (November), with an overall 1.24% annually. Wastage rates of PRBC, varied from 0.54% (January) to 1.68% (September), with an overall 1.06% annually. Wastage rates of RDP+SDP, varied from 0.00% (October) to 74.6% (January), with an overall 36.6% annually. Wastage rates of FFP, varied from 1.99% (November) to 5.9% (October), with an overall 3.4% annually. Wastage rates exceeded their respective benchmarks, suggesting the need for better demand forecasting and inventory management of blood product. The Turnaround Time for cross-match of blood (TAT) for emergency cross-match varied from 23.9 minutes (June) to 29.84 minutes (January), with an overall 27.29 minutes annually. The TAT for routine cross-match varied from 111.4 minutes (December) to 135.62 minutes (October), with an overall 121.72 minutes annually. The TAT consistently met the benchmark indicating efficient laboratory processes. Component QC failure parameters, including whole blood (WB), PRBC, RDP and FFP, varied across components but remained generally low, with an annual average of 4.98%. The Adverse Donor Reaction Rate varied from 0.00% to 0.14%with an overall 0.09% annually and remained below the benchmark of 2% annually, demonstrating the safety of the blood donation process. Donor deferral rates fluctuated and generally moving around the benchmark of 9.90-18.78%, with an annual average of 14.88%, demonstrating the stringent donor selection procedure for patient safety. Blood component issue varied across the year from 75.65 % (January) to 89.41% (July) with an annual average of 83.93%. The TTIs Outliers deviation beyond ±2SD was varied from 0.00% to 15.4% across the year with an annual of 5.68% for HIV, 5.11% for HBsAg and 4.55% for HCV. The LJ Chart prepared for HIV, HBsAg and HCV doesn't show any consecutive deviations beyond ± 2SD throughout the study period, confirming the reliability of TTI testing procedures. The department followed west guard rules for monitoring L. J. Charts. Instances of delayed transfusions beyond 30 minutes occurred, but the rate remained below the benchmark of 15% with an overall delay of 10.9% annually, indicating prompt administration of blood products to patients. Total blood component issue to blood collection and donationratio ranged from 1.22 to 2.19 across the months, with an annual average of 1.72, indicating efficient blood collection and utilization. Overall, while certain parameters demonstrated compliance with NABH standards, others highlighted areas for improvement in the healthcare facility's blood transfusion practices. These findings underscore the importance of ongoing quality assessment and continuous improvement initiatives to ensure patient safety and quality of care.

## Discussion:

The findings of this study provide valuable insights into the adherence of a healthcare facility to the National Accreditation Board for Hospitals & Healthcare Providers (NABH) standards in the context of blood transfusion services. The discussion focuses on the implications of the observed results and potential avenues for improvement in blood transfusion practice. The consistent compliance with the benchmarks for Transfusion Transmissible Infections (2.19%), including HIV (0.25%), HBsAg (0.97%), HCV (0.43%) and syphilis (0.55%) reflect the effectiveness of infection control measures implemented within the facility. Compared to our study, lower prevalence of TTIs (1.87%) was observed by Nikhil *et al.* [12], higher prevalence (3.39%) was observed by Gnanaraj *et al.* [1] and similar to Thakur *et al.* [13, 14] these results underscore the importance of rigorous screening protocols and stringent quality assurance procedures in minimizing the risk of transfusion-transmissible infections among recipients. The mean Acute Transfusion Reaction Rate (ATRR) recorded in this study was 0.36%. Compared to previous research, lower incidences of ATRR were noted by Bhandari *et al.* (0.25%) [15], Bhattacharya *et al.* (0.18%) [16], Vartak *et al.* (0.16%) [17] and Hariharan *et al.* (0.14%) [18]. Allergic reactions comprised a significant portion of ATRs, alongside non-hemolytic febrile transfusion reactions. The prospective introduction of leuko-depleted blood bag sets is under consideration, especially for patients with thalassemia and others requiring frequent blood transfusions [19]. The study findings indicate varying wastage rates (WR) across different blood components. Specifically, whole blood (WB) exhibited a WR of 1.24%, while packed red blood cells (PRBC) had a WR of 1.06%, random donor platelets (RDP) recorded a WR of 36.6% and fresh frozen plasma (FFP) showed a WR of 3.4%. Overall, when excluding Transfusion Transmissible Infections (TTIs) reactivity, the collective WR for blood and its derivatives was calculated at 11.48%. Comparatively, Gnanaraj *et al.* has reported a lower WR of 2.11% for blood and its derivatives, excluding TTI reactivity. Looking at regional variations, Nair *et al.* (2021), South India has reported overall WR of 6.14%. The WR for PRBC, plasma and platelets was 4.23%, 3.56%, and 16.6% respectively [20]. Similarly, Simon *et al.* (2020, South India) [21] observed a WR of 3.5% for PRBC, 5.5% for plasma and 52% for platelets, resulting in an overall WR of 19.3% for blood and its derivatives. Kanani *et al.* (2017, West India) [22] has reported a WR of 2.26% for PRBC, 5.36% for plasma and 28.39% for platelets, leading to an overall WR of 6.95% for blood and its derivatives. Lastly, Kumari *et al.* (2019, East India) [23] noted substantially higher WR percentages across all components, with 21.4% for PRBC, 11.7% for plasma and 66.4% for platelets, resulting in an overall WR of 22.8% for blood and its derivatives. Similar to present study results, other study shows higher wastage rate of platelet compared to other components due to short shelf life. The common causes of wastage of blood and its component are TTIs reactivity, outdated, lipemic blood, under collection and leakage or breakage of blood bag during processing. In the present study, the turnaround time (TAT) for emergency cross-match was found to be 27.29 minutes, while for Routine cross-match, it was notably higher at 121.74 minutes. In comparison, Gnanaraj *et al.* has reported a lower TAT of 18.3 minutes for both emergency and routine cases. However, Bhandari *et al.* has documented a TAT of 32.40 minutes for emergency cases and 148.6 minutes for routine cases. Similarly, Varshney and Gupta [8] reported TATs of 29.87 minutes for emergency cases and 135.82 minutes for routine cases.

Hariharan *et al.* [18] found TATs of 27.61 minutes for emergency cases and 143.56 minutes for routine cases, while Mukherjee *et al.* [24] reported TATs of 28.50 minutes for emergency cases and 141.38 minutes for routine cases. A root cause analysis of the higher TAT in our study revealed excessive workload and limited manpower at the blood bank, often necessitating the splitting of resources to serve both in-house and field camp services. Therefore, consideration should be given to recruiting more skilled technicians to alleviate this burden. Additionally, designating a senior technical staff member to supervise and manage operations during peak times can ensure the smooth functioning of the Blood Transfusion Service (BTS). With some variations the overall component QC failure was 5.09% for WB, 5.66% for PRBC and 8.62% FFP which is within the acceptable range. In comparison, Gnanaraj *et al.* [1] reported a higher component QC failure of 23.33% for PRBC, 21.67% for RDP and 41.11% for FFP. The findings related to Component Quality Control (QC) Failure parameters highlight the vigilant monitoring, staff training and adherence to standard operating procedures to maintain high- quality standards and mitigate potential risks to patient safety at our blood centre. In the present study, the Adverse Donor Reaction (ADR) rate was determined to be 0.09%, which was both comparable to and lower than the rates reported by Gnanaraj *et al.* [1] (1.71%), Bhandari *et al.* (1.26%) and Varshney and Gupta (1.15%). Among the ADR cases identified, the majority were attributed to vasovagal syncope, followed by hematoma occurrences. Notably, our observations underscore the pivotal role of pre- and post-donation counseling in mitigating the occurrence of adverse reactions among donors. In our study, the donor deferral rate was determined to be 14.88%, which falls within the range reported by previous studies. Specifically, it is comparable to rates reported by Gnanaraj *et al.* [1] (15.19%) and Bhandari *et al.* [15] (12.2%), which were similar to Agnihotri *et al.* [25] (11.6%) and Rehman *et al.* [26] (12.4%). However, some authors such as John and Varkey [27] (5.12%), Varshney and Gupta (9.3%) and Hari Haran *et al.* (8.99%) has reported comparatively lower deferral rates. The most common reason for deferral criteria observed across studies were low hemoglobin levels, engagement in high-risk activities or being unfit for blood donation due to other medical reasons. Notably, a significant proportion of deferred donors were found to be replacement donors. To address this issue, awareness initiatives were implemented aimed at increasing the recruitment of voluntary donors and promoting the conversion of replacement donors to voluntary status. In our investigation, we examined the Transfusion Transmissible Infection (TTIs) outliers' % by plotting LJ charts for both external and Internal (In-house) positive controls. The overall TTIs Outliers were found to be 5.68% for HIV, 5.11% for HBsAg and 4.55% for HCV. These rates are comparable to those reported by Gnanaraj *et al.* [1] who observed TTI Outliers of 14.43% for HBsAg, 12.59% for HCV and 17.73% for HIV. Despite the detection of TTI Outliers beyond ± 2SD, our department adhered to West Guard rules for monitoring LJ Charts. Analysis of the LJ Chart data for HIV, HBsAg and HCV outliers revealed no consecutive deviations beyond ± 2SD throughout the study period, thus confirming the reliability of our TTI testing procedures. Our investigation found a 10.9% Delay in Transfusion beyond 30 minutes after issue, lower than Gnanaraj *et al.* [1] 19.14%. Delays were often due to unavailability of nursing or medical staff. Training sessions were held to educate staff on proper transfusion procedures and audits were conducted to ensure adherence to protocols. The present study shows that 83.93% of the total blood and blood component issued. Additionally, a new Key Performance Indicator (KPI) was introduced - the total blood component issue to blood collection ratio. In our study, this ratio was calculated at 1.72, indicating that collected blood units were utilized 1.72 times. These findings underscore the significance of our study in assessing the effectiveness of blood component preparation and utilization similar to other studies [28, 29]. Overall, the results of this study underscore the importance of continuous quality improvement initiatives in blood transfusion services using manual KPI-dashboard. Addressing the identified gaps and building upon existing strengths can contribute to the enhancement of patient outcomes, optimization of resource utilization and alignment with international quality standards in healthcare delivery. Future research could focus on implementing targeted interventions and evaluating their impact on improving compliance with NABH standards and enhancing the overall quality of blood transfusion services.

## Conclusion:

Overall, continuous quality improvement initiatives are crucial in blood transfusion services to enhance patient outcomes and align with international quality standards. KPI-Dashboard is very helpful in identifying the current lacunae which can be very helpful in improving blood transfusion services. Future research could focus on implementing targeted interventions to improve compliance with NABH standards and enhance overall quality in transfusion practice. Present study describes a concept of manual dashboard. A new KPI was also introduced in the present study.

## Author contributions:

The research design was developed by Thakur SK, Kumar A, Fazil M, Sharma SK, Kumar L, Jahan A, Gupta R and Singh S. Thakur SK carried out the literature review, data collection, data analysis and manuscript preparation. Thakur SK, Gupta R and Singh S performed Statistical analysis. All authors equally contributed to data processing, interpretation, writing and revising the final text.

## Supported by:

This study received no specific funding from government, commercial, or non-profit sources.
